# Prevalence and gender differences in symptomatology of posttraumatic stress disorder and depression among Iraqi Yazidis displaced into Turkey

**DOI:** 10.3402/ejpt.v7.28556

**Published:** 2016-02-12

**Authors:** Atilla Tekin, Hekim Karadağ, Metin Süleymanoğlu, Merve Tekin, Yusuf Kayran, Gökay Alpak, Vedat Şar

**Affiliations:** 1 Department of Psychiatry, Cizre State Hospital, Şırnak, Turkey; 2 Department of Psychiatry, Van Regional Training and Educational Hospital, Van, Turkey; 3 Department of Neurology, İdil State Hospital, Şırnak, Turkey; 4 Department of Psychiatry, Gaziantep University School of Medicine, Gaziantep, Turkey; 5 Department of Psychiatry, Koç University School of Medicine, Istanbul, Turkey

**Keywords:** Depression, posttraumatic stress disorder, refugee, gender-based differences, traumatic life events

## Abstract

**Background:**

Posttraumatic stress disorder (PTSD) and depression are common among populations displaced due to large-scale political conflicts and war.

**Objective:**

The aim of this study is to investigate the prevalence and gender-based differences in symptoms of PTSD and depression among Iraqi Yazidis displaced into Turkey.

**Method:**

The study was conducted on 238 individuals who were evaluated using the Structured Clinical Interview for DSM-IV (SCID-I) and the Stressful Life Events Screening Questionnaire.

**Results:**

Of the participants, 42.9% met the DSM-IV diagnostic criteria for PTSD, 39.5% for major depression, and 26.4% for both disorders. More women than men suffered from PTSD and major depression. More women than men with PTSD or depression reported having experienced or witnessed the death of a spouse or child. Women with PTSD reported flashbacks, hypervigilance, and intense psychological distress due to reminders of trauma more frequently than men. Men with PTSD reported feelings of detachment or estrangement from others more frequently than women. More depressive women than men reported feelings of guilt or worthlessness.

**Conclusions:**

PTSD and major depression affected women more frequently than men. While women tended to respond to traumatic stress by undermodulation of emotions and low self-esteem, men tended to respond by overmodulation of emotions. Rather than being a derivative of sex differences, this complementary diversity in response types between genders seems to be shaped by social factors in consideration of survival under extreme threat.

Middle-eastern countries have increasingly been exposed to war and violent events due to long-lasting political conflicts. These events caused millions of people to abandon the places they used to live. Psychiatric disorders related to stress are known to be common in such populations alongside general health problems. Previous studies on refugees reported diverse prevalences of posttraumatic stress disorder (PTSD) (3–86%) and depression (3–80%) (Başoğlu et al., 2005; Fazel, Wheeler & Danesh, 2005; Steel et al., 2009).

A large population has been “displaced” and have taken shelter in Turkey following acts of war and conflict in the neighboring countries. The massive scale of this “displacement” phenomenon also shows certain different characteristics than “refuge” situations resulting from the relocating of hundreds of thousands of people in a very short time period. One of these communities has been the Yazidis of northern Iraq who had to move to Turkey in the summer of 2014 due to atrocities by the Islamic State of Iraq and Syria (ISIS), a terrorist organization. The main terrorist attack was conducted in the Shengal region. The migrants were settled in Cizre district of southeastern Turkey temporarily.

Yazidis, who are one of the oldest ethnic communities of Mesopotamia, mainly live in northern Iraq. Yazidism is a verbally transmitted religion and an important part of Kurdish folk culture. Even though Yazidis have been influenced by other religions such as Islam and Christianity, they have maintained their own unique religious beliefs throughout history. A major part of Iraqi Yazidis speak Kurdish, especially the Kurmanji dialect (Fuccaro, 1997).

The aim of this study was to investigate the prevalence of PTSD and depression in Iraqi Yazidi refugees who were displaced into Turkey due to the recent attacks. Gender differences in symptoms and antecedents of PTSD and depression have been the focus of this study. Gender differences in response to traumatic stress have been an important issue in studies on PTSD (Kimerling, Quimette, & Wolfe, 2002). While both genders are exposed to stressful events to a similar degree, twice as much women develop PTSD in response to these experiences (Norris, Foster, & Weisshaar, 2002). As type of traumatic event, women report sexual abuse more frequently and experiences of war are more prevalent among men. However, the population evaluated in the present study represents a rather homogenous pattern in terms of the overall traumatic stress they have been exposed to.

## Method

### Participants

The present study was conducted on migrants who were displaced from the Shengal region in Iraq and entered Turkey in a period as short as 3 months, that is, between July and September 2014. A representative study sample was selected from migrants (aged 18–65 years) who were staying at a camp during the study period (February–April 2015). This camp was located at the Cizre district near the Iraqi border of Turkey, and was built by the local municipal authority.

Demographic data on Yazidi migrants were obtained from Cizre Municipality. Among 3,413 inhabitants of the camp, 622 individuals were aged between 18 and 65 years. For the present study, a sample size calculation was based on error margin of 5%, 95% confidence level for a population of 622 individuals. The expected ratio of PTSD of 50% was run at the sample size calculator website (www.raosoft.com/samplesize.html) that resulted at 238. Participants were selected randomly using a computer-based random number generation (www.stattrek.com/statistics/random-number-generator.aspx).

Four individuals were unable to participate in the study due to physical disability (*n*=3) or severe mental retardation (*n*=1). Others of the same sex and age replaced them randomly. Each participant signed an informed consent form. None of the participants rejected participation. Ethical approval for the study was obtained from the Gaziantep University Ethics Committee.

### Assessment instruments

Demographical data, including age, sex, education level, marital status, occupation, duration of displacement, smoking status, alcohol intake, drug abuse, and psychiatric and other medical information, were collected using a sociodemographic history form.

#### Structured clinical interview for DSM-IV axis I disorders

The Structured Clinical Interview (SCID-I) was developed by First, Spitzer, Gibbon, and Williams (1997) according to DSM-IV (American Psychiatric Association, 1994) diagnostic criteria. Validity studies in Turkey were performed by Çorapçıoğlu, Aydemir, Yıldız, Esen, and Köroğlu (1999). PTSD and depression modules of the SCID-I were applied by direct translation of the questions into Kurdish by the interviewers.

#### Stressful life events screening questionnaire

This form was developed by Stamm and Rudolph in 1996. It is a self-report measure consisting of 20 questions that assesses lifetime exposure to traumatic events (Stamm, 1996). Each question in this scale evaluates the presence of a traumatic life event. Stressful life events screening questionnaire was translated into Kurdish by the research team before the interviews were conducted. A reliability or validity study does not exist for this particular screening instrument.

### Interviewing

Each participant was informed about the purpose of the study to get consent. After administration of the sociodemographic data collection, the PTSD and depression modules of the SCID-I were conducted. Potentially traumatic life events were screened afterwards. Two male psychologists with clinical experience (HK and MS) who grew up in the region and were fluent in both Turkish and Kurdish administered the assessment tools separately. Each interview was conducted by a psychologist privately in a room located inside the camp. The interviewers had previous experience in application of SCID-I. They were additionally trained by the principal investigator (AT), a psychiatrist, about the general frame of the interview. Since there was no Kurdish version of the SCID-I available, the interviewers had to formulate the questions in Kurdish while remaining loyal to the Turkish text. The importance of using identical Kurdish expressions for the questions throughout clinical interviews was underlined to maintain uniformity.

### Statistical analysis

Descriptive statistics were given as frequency, percentage, mean, standard deviation, and minimum–maximum values. Differences between categorical variables in the groups were analyzed with the Chi-square test. Normality assessment of the continuous variables was performed with Kolmogorov–Smirnov and Shapiro–Wilk test. Comparisons of the variables that fitted normal distribution were evaluated with the Student's *t*-test. Comparisons of the variables that do not show normal distribution were made with Mann–Whitney U test.

## Results

Mean age of the participants was 32.70±11.87 (range=18–65) and mean duration of the displacement was 3.87±0.51 months (range=3–5 months). Among 238 participants, 133 were female (55.9%). There was no significant difference between women (mean=31.91, SD=11.69) and men (mean=33.70, SD=12.06) on age (*t*=1.16, df=236, *p*=0.247).

A total of 102 (42.9%) participants met the diagnostic criteria for current PTSD and 94 (39.5%) for current major depression. Sixty-three participants (26.4%) had both conditions. A total of 123 (51.7%) participants met the diagnostic criteria for lifetime PTSD and 110 (46.2%) had a lifetime diagnosis of major depression. Among those with current PTSD, the disorder was chronic for 96 (94.2%) participants. Thirty-six (15.1%) participants had a history of at least one previous psychiatric admission.

Of the 102 participants with current PTSD, 76 (74.5%) were female (*χ* ^2^=25.12, df=1, *p*=0.001). This rate was 78.7% (*n*=74) for women (*χ* ^2^=32.88, df=1, *p*=0.001) with current major depression (*n*=94). There was no difference in age between women with (mean=32.87, SD=11.97) and without PTSD (mean=30.63, SD=11.29) (*t*=1.09, df=100, *p*=0.277). The duration of displacement between women with (mean=3.86, SD=0.58) and without PTSD (mean=3.95, SD=0.58) (*z*=0.81, *p*=0.418) did not differ either. There was also no difference on educational status (*χ* ^2^=3.96, df=4, *p*=0.412) and rate of psychiatric admission in the past between women with (26.3%) and without (17.5%) PTSD (*χ* ^2^=1.44, df=1, *p*=0.230).

Women with depression were older (*n*=74, 55.6%, mean=34.72, SD=12.70) than those without (*n*=59, 44.4%, mean=28.39, SD=9.26) (*t*=3.21, df=131, *p*=0.002). The duration of displacement did not differ between women with (mean=3.88, SD=0.60) and without depression (mean=3.92, SD=0.57) (*z*=0.27, *p*=0.788). There was no difference on educational status between two groups (*χ* ^2^=2.026, df=4, *p*=0.155) either. The rate of psychiatric admission in the past was higher among women with depression (31.1%) than those without (11.9%) (*χ* ^2^=6.94, df=1, *p*=0.008).

More women (*n*=102, 76.7%) than men (*n*=68, 64.8%) had been in a region that was affected by war (*χ* ^2^=4.09, df=1, *p*=0.043), experienced/witnessed an attack with a weapon (*n*=95, 71.4% vs. *n*=61, 58.1%) (*χ* ^2^=4.17, df=1, *p*=0.032), witnessed and touched dead bodies apart from funerals (*n*=46, 34.6% vs. *n*=21, 20.0%) ( *χ* ^2^=6.17, df=1, *p*=0.013), and witnessed someone else being choked, spanked, or pushed (*n*=43, 32.3% vs. *n*=21, 20.0%) (*χ* ^2^=4.54, df=1, *p*=0.033).

There was no difference in age between men with (mean=34.50, SD=11.73) and without PTSD (mean=33.44, SD=12.23) (*t*=0.39, df=100, *p*=0.700). The duration of displacement did not differ between men with (mean=3.85, SD=0.46) and without PTSD (mean=3.84, SD=0.37) (*z*=0.05, *p*=0.960) either. Two groups did not differ on educational status (*χ* ^2^=1.95, df=4, *p*=0.745). The rate of psychiatric admission in the past was higher among men with PTSD (19.2%) than those without (1.3%) (Fischer's exact test, *p*=0.003).

There was no difference in age between men (*n*=20, 19.0%) with (mean=31.30, SD=10.51) and without (*n*=85, 81.0%) depression (mean=34.27, SD=12.39) (*t*=0.99, df=103, *p*=0.324). The duration of displacement did not differ between men with (mean=3.95, SD=0.39) and without depression (mean=3.81, SD=0.39) (*z*=1.34, *p*=0.181). The two groups did not differ on educational status either (*χ* ^2^=7.65, df=4, *p*=0.105). The rate of psychiatric admission in the past was higher among depressive men (25.0%) than those without depression (1.2%) (Fischer's exact test, *p*=0.001).

Table 1 demonstrates a comparison of traumatic events reported by women with or without PTSD or depression. Among women, while three items were elevated in both diagnostic groups (having experienced or witnessed the death of a spouse/child or a close friend or a family member and having witnessed and touched dead bodies except at funerals), the following three items were elevated in the PTSD group only: having experienced/witnessed a terrorist attack or torture, having been in a region that is affected by war, and having experienced/witnessed an attack with a weapon.

**Table 1:** Comparison of traumatic life events in women with or without PTSD or depression

	With PTSD		Without PTSD		*χ*^2^		With depression		Without depression		*χ*^2^	
				
Traumatic life events	*n*=76	*n* %	*n*=57	*n* %	(df=1)	*p*	*n*=74	*n* %	*n*=59	*n* %	(df = 1)	*p*
Experienced/witnessed a natural disaster	23	30.3	20	35.1	0.35	0.556	24	32.4	19	32.2	0.00	0.978
Experienced/witnessed a serious accident or injury	13	17.1	8	14.0	0.23	0.631	14	18.9	7	11.9	1.23	0.268
Experienced/witnessed a life-threatening disease	10	13.2	4	7.0	1.30	0.253	10	13.5	4	6.8	1.58	0.209
Experienced/witnessed the death of a spouse/child	14	18.4	0	0.0	11.74	0.001	12	16.2	2	3.4	5.73	0.017
Experienced/witnessed death of a close friend or a family member (except spouse/child)	17	22.4	1	1.8	11.83	0.001	14	18.9	4	6.8	4.13	0.042
Experienced/witnessed a terrorist attack or torture	10	13.2	1	1.8		0.024^a^	5	6.8	6	10.2		0.537^a^
Had been in a region that is affected by war	69	90.8	33	57.9	19.72	0.001	59	79.7	43	72.9	0.86	0.353
Witnessed and touched dead bodies apart from funerals	40	52.6	6	10.5	25.52	0.001	32	43.2	14	23.7	5.53	0.019
Experienced/witnessed an attack with a weapon	72	94.7	23	40.4	47.21	0.001	57	77.0	38	64.4	2.56	0.109
Experienced being spanked, choked, or pushed as a child/teen	28	36.8	15	26.3	1.65	0.199	21	28.4	22	37.3	1.19	0.275
Experienced being spanked, choked, or pushed as an adult	11	14.5	3	5.3	2.93	0.087	5	6.8	9	15.3	2.52	0.113
Witnessed someone else being choked, spanked, or pushed	29	38.2	14	24.6	2.75	0.097	24	32.4	19	32.2	0.00	0.978
Witnessed someone else being forced to have unwanted sexual contact	2	2.6	1	1.8		1.0^a^	2	2.7	1	1.7		1.0^a^

a Fischer's Exact test. *χ*^2^: Pearson Chi-square test, df: degree of freedom.

Among men, following two traumatic experiences were elevated in both PTSD and depressive disorder: Having experienced/witnessed death of a close friend or a family member (except spouse/child), and having witnessed and touched dead bodies apart from funerals. Unlike for the depression group, the following two items were elevated in the PTSD group only: Having been in a region that is affected by war and having experienced/witnessed an attack with a weapon.

Unlike for women, having experienced/witnessed a terrorist attack or torture was not elevated in the male group with PTSD. Both for PTSD and depression, one item was elevated among women only (Table 2): *having experienced or witnessed the death of a spouse or child*. Women with PTSD reported flashbacks, intense psychological distress, and hypervigilance more frequently than men (Table 3). However, “feelings of detachment or estrangement from others” were more common among men. Among depressive symptoms, guilt/worthlessness was more frequent among women than men (Table 4).

**Table 2:** Comparison of traumatic life events in men with or without PTSD or depression

	With PTSD		Without PTSD		*χ*^2^		With depression		Without depression		*χ*^2^	
				
Traumatic life events	(*n*=26)	*n* %	(*n*=79)	*n* %	(df = 1)	*p*	(*n*=20)	*n* %	(*n*=85)	*n* %	(df = 1)	*p*
Experienced/witnessed a natural disaster	9	34.6	31	39.2	0.18	0.674	8	40.0	32	37.6	0.04	0.845
Experienced/witnessed a serious accident or injury	8	30.8	13	16.5	2.51	0.113	5	25.0	16	18.8		0.542^a^
Experienced/witnessed a life-threatening disease	1	3.8	3	3.8		1.0^a^	1	5.0	3	3.5		0.576^a^
Experienced/witnessed the death of a spouse/child	3	11.5	2	2.5		0.096^a^	2	10.0	3	3.5		0.241^a^
Experienced/witnessed death of a close friend or a family member (except spouse/child)	6	23.1	2	2.5		0.003^a^	5	25.0	3	3.5		0.006^a^
Experienced/witnessed a terrorist attack or torture	1	3.8	2	2.5		1.0^a^	0	0.0	3	3.5		1.0^a^
Had been in a region that is affected by war	24	92.3	44	55.7	11.49	0.001	13	65.0	55	64.7	0.00	0.980
Witnessed and touched dead bodies apart from funerals	13	50.0	8	10.1	19.44	0.001	9	45.0	12	14.1		0.004^a^
Experienced/witnessed an attack with a weapon	23	88.5	38	48.1	13.09	0.001	11	55.0	50	58.8	0.10	0.755
Experienced being spanked, choked, or pushed as a child/teen	10	38.5	20	25.3	1.66	0.198	4	20.0	26	30.6	0.89	0.346
Experienced being spanked, choked, or pushed as an adult	2	7.7	8	10.1		1.0^a^	3	15.0	7	8.2		0.398^a^
Witnessed someone else being choked, spanked, or pushed	5	19.2	16	20.3	0.01	0.910	3	15.0	18	21.2		0.758^a^
Witnessed someone else being forced to have unwanted sexual contact	1	3.8	0	0.0		0.248^a^	1	5.0	0	0.0		0.190^a^

a Fischer's Exact test. *χ*^2^: Pearson Chi-square test, df: degree of freedom.

**Table 3:** Comparison of symptoms in women and men with PTSD

	Women		Men			
				
Symptoms	(*n*=76)	*N* %	(*n*=26)	*n* %	*χ* ^2^ (df = 1)	*p*
B1.Reccurent/intrusive recollecting	61	80.5	22	84.6		0.774^a^
B2.Recurrent distressing dreams	47	61.8	13	50.0	1.12	0.290
B3.Flashbacks	35	46.1	6	23.1	4.25	0.039
B4.Intense psychological distress	46	60.5	8	30.8	6.89	0.009
B5.Physiologic reactivity	44	57.9	18	69.2	1.04	0.307
C1.Efforts to avoid thoughts, feelings, or conversations	37	48.7	9	34.6	1.55	0.213
C2.Efforts to avoid activities, places, or people	7	9.2	2	7.7		1.0^a^
C3.Inability to recall an important aspect of the trauma	25	32.9	6	23.1	0.88	0.347
C4.Diminished interest or participation in significant activities	49	64.5	18	69.2	0.20	0.659
C5.Feeling of detachment or estrangement from others	49	64.5	23	88.5	5.37	0.020
C6.Restricted range of affect	61	80.3	19	73.1	0.59	0.442
C7.Sense of foreshortened future	70	92.1	24	92.3		1.0^a^
D1.Difficulty falling or staying asleep	40	52.6	14	53.8	0.01	0.915
D2.Irritability or outbursts of anger	28	36.8	14	53.8	2.31	0.128
D3.Difficulty concentrating	24	31.6	5	19.2	1.45	0.228
D4.Hypervigilance	67	88.2	16	61.5	9.06	0.007
D5.Exaggerated startle response	25	32.9	8	30.8	0.04	0.842

a Fischer's Exact test. *χ*^2^: Pearson Chi-square test, df: degree of freedom.

**Table 4:** Comparison of symptoms in women and men with depression

	Women		Men			
				
Symptoms	(*n*=74)	*n* %	(*n*=20)	*n* %	*χ* ^2^ (df = 1)	*p*
A1.Depressed mood	70	94.6	19	95.0		1.0^a^
A2.Decreased interest or pleasure	68	91.9	17	85.0		0.395^a^
A3.Significant appetite/weight change	51	68.9	13	65.0	0.11	0.739
A4.Change in sleep	48	64.9	17	85.0	2.99	0.084
A5.Psychomotor agitation or retardation	42	56.8	10	50.0	0.29	0.590
A6.Fatigue or loss of energy	52	70.3	11	55.0	1.66	0.197
A7.Guilt/worthlessness	58	78.4	11	55.0	4.41	0.036
A8.Concentration	57	77.0	17	85.0		0.550^a^
A9.Suicidality	35	47.3	6	30.0	1.92	0.166

a Fischer's Exact test. *χ*^2^: Pearson Chi-square test, df: degree of freedom.

## Discussion

Prevalence of PTSD and depression reported by studies conducted on refugees varies considerably. In a systematic review (Hollifield et al., 2002), the prevalence of PTSD and depression was reported as 4–86% and 5–31%, respectively. These figures were 5.6 and 18.5% for Bosnian refugees (Vuković, Jovanović, Kolarić, Vidović, & Mollica, 2014). For Iraqi refugees living in western countries, this rate varied between 8 and 37.2% for PTSD and between 28.3 and 75% for depression (Slewa-Younan, Uribe Guajardo, Heriseanu, & Hasan, 2014; Sondergaard, Ekblad, & Teorell, 2001). In post-conflict communities of Algeria, Cambodia, Ethiopia, and Palestine, the rates of PTSD and mood disorders were between 15.8–37.4% and 5.2–22.7%, respectively (De Jong, Komproe, & Van Ommeren, 2003). In an adult population directly exposed to war in the Balkans, the prevalence of PTSD and depression was between 10.6–35.4% and 10.9–37.3%, respectively (Priebe et al., 2010). Among secondary school students in Baghdad, the prevalence of PTSD increased even to 61% (Al-Hadethe, Hunt, Thomas, & Al-Qaysi, 2014).

Representing a rather moderate figure among all studied populations, 4 of 10 participants in the present study had either PTSD or depression. Tufan, Alkin, and Bosgelmez (2013) reported the prevalence of PTSD and depression in refugees living in Turkey as 55.2 and 55.2%, respectively. In another study conducted among Syrian refugees in Turkey (Alpak et al., 2015), the prevalence of PTSD was 33.5%. Thus, the few currently existing studies on populations displaced into Turkey reported rates close to each other. Among others, this may be due to the homogeneity of the displacement duration among studied populations. Indeed, the recent crisis at the borders of Turkey and the subsequent massive displacement happened in a relatively short time period.

The differences among symptoms between women and men with PTSD and depression are rather consistent in fitting certain patterns of posttraumatic response. Namely, women reported flashbacks, hypervigilance, and intense psychological distress due to reminders of trauma more frequently than men. Men reported feelings of detachment or estrangement from others more frequently than women. Thus, women tended to respond to traumatic stress by undermodulation and men tended to respond by overmodulation of emotions (Lanius et al., 2010). Representing a rather avoidant type of strategy, emotional overmodulation is related to the dissociative subtype of PTSD as defined in DSM-5 (American Psychiatric Association, 2013). Namely, this subtype is characterized by depersonalization and/or derealization. Representing a rather intrusion type of response, emotional undermodulation involves the predominance of re-experiencing and hyperarousal symptoms.

A number of neurobiological differences have been found between these two types of PTSD. Hyperinhibition of the limbic regions by medial prefrontal areas was shown with emotional overmodulation, while the failure of prefrontal inhibition of limbic regions was found with emotional undermodulation (Bremner, 1999; Lanius et al., 2010). While emotional overmodulation may assist in coping with extraordinary conditions at least in early stages of the response, emotional undermodulation may serve as a “call for rescue.” It is unknown whether the radical distribution of these two response types observed in the studied group is associated with sex (biologic factors), gender (social factors), or a mix of both.

When facing a vital threat, fight, flight, submission, and freezing are psychobiological action types that help individuals to survive (Van der Hart, Nijenhuis, & Steele, 2006). Leaving a conflict zone in search of a safer place is an indicator of “flight”-type response, undermodulation of emotions usually being a part of this response, whereas overmodulation is rather associated with “submission” and “freezing.” In contrast to the conventional gender stereotypes, symptoms that are traditionally associated with the “fighting” role have been predominantly observed in the female participants of this study, whereas the male participants reflected an overmodulation type of response associated with “submission” or “freezing” type of action. However, these results may also be a selection bias. The study examines a displaced population consisting of individuals who have inherently shown a flight-type of action as a matter of course. Indeed, many males with a predominantly fight-type reflex may have refused to show a flight-type of response and chosen to stay in the conflict zone. The study does not include an examination of these individuals. As a result, some of these males may have even failed to survive as a consequence of their predominantly fight-type response.

In most of the middle-eastern countries, polarized gender stereotypes are common to devote a rather “passive” role to women while men have to be socially dominant. However, in wartime and periods of conflict, this predefined gender-role poses an inherent risk for men as “fight” by brute force is more readily justified, or becomes even a duty. Hence, unlike for women, a “submissive” role for men may additionally provide relative safety in avoiding violent engagements under conditions of wide-scale power conflict. From a psychosocial perspective, taking differences in gender roles into consideration, the duality in the response types of the studied population seems to serve a “family balance” not only in terms of collaboratively processing the emotions but also in acting for survival as a small group.

In the present study, more women than men suffered from PTSD and major depression. Interestingly, more women than men reported being exposed to war-related events such as having been in a region that was affected by war; experienced/witnessed an attack with a weapon; witnessed and touched dead bodies apart from funerals; and witnessed someone else being choked, spanked, or pushed. This observation also supports the presence of a sampling bias reported above.

Women with depression reported guilt and worthlessness more frequently than men. Sar, Akyüzü, Öztürk, and Alioğlu (2013) reported preponderance of feelings of guilt and worthlessness among women with dissociative depression compared with those without dissociation. Both dissociative depression and the dissociative subtype of PTSD are known to be related to chronic childhood trauma as seen in Complex PTSD (Sar, 2011). A number of studies have examined the dissociative experiences of refugees (Douglas, 1992; Marmar et al., 1994; Punamäki, Komproe, Qouta, Elmasri, & De Jong, 2005). Marmar et al. (1994) found a relationship between peritraumatic dissociation and a PTSD diagnosis in male Vietnam theater veterans. In a sample of 316 Vietnam veterans, Waelde, Silvern, and Fairbank (2005) found a taxon of highly dissociative individuals.

The preponderance of guilt and worthlessness may also be associated with the type of traumatic events. Namely, both in the PTSD and depression groups, more women reported having experienced or witnessed the death of a spouse or a child. Being killed during war seems to be more likely for children and men. While the latter may occur due to a direct target in combat, the former seems to occur due to greater vulnerability in dangerous situations.

On the contrary, unlike in men, the results show a greater exposure to terrorist attacks or torture among the female group with PTSD. Women may have been more vulnerable to these experiences compared to men in the studied group possibly due to “survivor's guilt” (e.g., in response to loss of family members or having left others behind) among possible other factors. Unlike men and participants of both genders with PTSD, women with depression were older than those without.

It is of particular interest that, in the present study, both men and women with depression (but not those with PTSD) reported previous psychiatric treatment more frequently than those without. These findings may point to better access of depressive subjects to mental health services in their country of origin compared with those with PTSD. Typically, PTSD is less recognized than depression both by mental health professionals and by people suffering from this condition. This may be partly related to the higher prevalence of depression than PTSD in the general population. On the contrary, a past episode of depression is a well-known risk factor for a future depressive attack due to diverse reasons covering both environmental and constitutional factors.

In the present study, PTSD was chronic for 94.2% of the affected participants. In a group of Syrian refugees in Turkey (Alpak et al., 2015), the condition was chronic for 89.0% of the participants with PTSD. Acute PTSD affected only 9.3% and late onset PTSD affected only 1.7% of this group. While depression may also develop as a complication of chronic PTSD, the latter should be screened in displaced individuals who apply to mental health services due to depressive symptoms. Their psychotherapeutic and psychopharmacological treatment may differ from those who come up with a “primary” depressive disorder.

There were several limitations to the present study. First, screening was done only in one out of three camps where Iraqi Yazidis were staying. Also, there were no assessment instruments available in the native language of the participants. However, the urgency of the situation made these efforts valuable in terms of approaching an understudied population. The importance of the present study is in the evaluation of gender and sex differences in posttraumatic response in a study group who were exposed to similar stress factors with a limited set of differences. However, different universal psychobiological action systems of survival seem to prevail among men and women (in a complementary fashion) in this early period of extreme stress which is assumed to be socially determined rather than representing biologically anchored sex differences.

The findings of the present study may have implications for treatment as well. Namely, the distinct types of posttraumatic reaction may respond to different treatment modalities (Brown, 2006). On the contrary, post-migration experiences, such as unemployment, insecure residency, fear of repatriation, and social discrimination are also related to mental problems in refugees (Bemak & Chung, 2014; Newman, 2013). Hence, better social integration may be expected in conditions where similarities exist between sociocultural features of the host country and the country of origin. However, this needs to be proven by evidence-based research. Last but not least, this study documents features of response to stress among displaced people in early phases of migration. Future research, in particular follow-up studies, would illuminate the outcome of the different types of response to traumatic stress among men and women, as well as their response to diverse treatment modalities.
